# Editorial: Spiking Neural Network Learning, Benchmarking, Programming and Executing

**DOI:** 10.3389/fnins.2020.00276

**Published:** 2020-04-15

**Authors:** Guoqi Li, Lei Deng, Yansong Chua, Peng Li, Emre O. Neftci, Haizhou Li

**Affiliations:** ^1^Department of Precision Instrument, Center for Brain Inspired Computing Research, Tsinghua University, Beijing, China; ^2^Beijing Innovation Center for Future Chips, Tsinghua University, Beijing, China; ^3^Department of Electrical and Computer Engineering, University of California, Santa Barbara, Santa Barbara, CA, United States; ^4^Huawei Technologies, Shenzhen, China; ^5^Department of Cognitive Science, University of California, Irvine, Irvine, CA, United States; ^6^Department of Electrical Engineering, National University of Singapore, Singapore, Singapore

**Keywords:** deep spiking neural networks, SNN learning algorithms, programming framework, SNN benchmarks, neuromorphics

## Introduction

A spiking neural network (SNN), a type of brain-inspired neural network, mimics the biological brain, specifically, its neural codes, neuro-dynamics, and circuitry. SNNs have garnered great interest in both Artificial Intelligence (AI) and neuroscience communities given its great potential in biologically realistic modeling of human cognition and development of energy efficient, event-driven machine learning hardware (Pei et al., 2019; Roy et al., 2019). Significant progress has been made across a wide spectrum of AI fields, such as image processing, speech recognition, and machine translation. They are largely driven by the advance in Artificial Neural Networks (ANN) in systematic learning theories, explicit benchmarks with various tasks and data sets, friendly programming tools [e.g., TensorFlow (Abadi et al., 2016) and Pytorch (Paszke et al., 2019) machine learning tools], and efficient processing platforms [e.g., graphics processing unit (GPU) and tensor processing unit (TPU) (Jouppi et al., 2017)]. In comparison, SNNs are still at an early stage in these aspects. To further exploit the advantages of SNNs and attract more researchers to contribute in this field, we proposed a Research Topic in Frontiers in Neuroscience to discuss the main challenges and future prospects of SNNs, emphasizing on its “Learning algorithms, Benchmarking, Programming, and Executing.” We are confident that SNNs will play a critical role in the development of energy efficient machine learning devices through algorithm-hardware co-design.

This Research Topic brings together researchers of different disciplines in order to present their recent work in SNNs. We received 22 submissions worldwide and accepted 15 papers. The scope of the accepted papers covers learning algorithms, model efficiency, programming tools, and neuromorphic hardware.

## Learning Algorithms

Learning algorithms play perhaps the most important role in AI techniques. Machine learning algorithms, in particular those for deep neural networks (DNN), have become the standard bearer in a wide spectrum of AI tasks. Some of the more common learning algorithms include backpropagation (Hecht-Nielsen, 1992), stochastic gradient descent (SGD) (Bottou, 2012), and ADAM optimization (Kingma and Ba, 2014). Other techniques such as batch normalization (Ioffe and Szegedy, 2015) and distributed training (Dean et al., 2012) facilitate learning in DNNs and enable them to be applied in various real-world applications. In comparison, there are relatively fewer SNN learning algorithms and techniques. Existing SNN learning algorithms fall into three categories: unsupervised learning algorithms such as the original spike timing dependent plasticity (STDP) (Querlioz et al., 2013; Diehl and Cook, 2015; Kheradpisheh et al., 2016), indirect supervised learning such as ANN-to-SNN conversion (O'Connor et al., 2013; Pérez-Carrasco et al., 2013; Diehl et al., 2015; Sengupta et al., 2019), and direct supervised learning such as spatiotemporal backpropagation (Wu et al., 2018, 2019a,b). We note that progress in STDP research includes introducing a reward or supervision signal such as spike timing which, in combination with this third factor, dictates the weight changes (Paugam-Moisy et al., 2006; Franosch et al., 2013). Despite the progress made, no algorithm can yet train a very deep SNN efficiently, which has become almost the holy grail of our field. Below, we briefly summarize the accepted algorithm papers in this Research Topic.

Inspired by the mammalian olfactory system, Borthakur and Cleland develop an SNN model trained using STDP for signal restoration and identification. It is broadly applicable to sensor array inputs. Luo et al. propose a new weight update mechanism that adjusts synaptic weights, leading to the first wrong output spike-timing to classify input spike trains with time-sensitive information accurately. He et al. divide the learning (weight training) process into two phases: the structure formation phase using Hebb's rule, and the parameter training phase using STDP and reinforcement learning, so as to form an SNN-based associative memory system. In contrary to just training synaptic weights, Wang et al. propose training both the synaptic weights and delays using gradient descent so as to achieve better performance. Based on a structurally fixed small SNN with sparse recurrent connections, Ponghiran et al. use Q-learning to train only its output layer so as to achieve human-level performance on complex reinforcement learning tasks such as Atari games. Their research demonstrates that a small random recurrent SNN is able to provide a computationally efficient alternative to state-of-art deep reinforcement learning networks with several layers of trainable parameters. The above works have made good progress toward better performing SNN learning algorithms. We believe that further progress will be made in this field in the future.

## Model Efficiency

In recent years, hardware oriented DNN compression techniques have been proposed that offer significant memory saving and hardware acceleration (Han et al., 2015a, 2016; Zhang et al., 2016; Huang et al., 2017; Aimar et al., 2018). At present, many compression techniques are proposed that provide a trade-off between processing efficiency and application accuracy (Han et al., 2015b; Novikov et al., 2015; Zhou et al., 2016). Such an approach has also caught on in the design of SNN accelerators (Deng et al., 2019), with the following contribution in this Research Topic. Afshar et al. investigate how a hardware-efficient variant of STDP may be used for event-based feature extraction. Using a rigorous testing framework, a range of spatio-temporal kernels with different surface decaying methods, decay functions, receptive field sizes, feature numbers, and backend classifiers are evaluated. This detailed investigation provides useful insight and heuristics with regards to the trade-off between performance and complexity while using the STDP rule. Pedroni et al. study the impact of different arrangements of synaptic connectivity tables on weight storage and STDP updates for large-scale neuromorphic systems. Based on their analysis, they present an alternative formulation of STDP via a delayed causal update mechanism that permits efficient weight storage and access for both full and sparse connectivity.

Other than model complexity, several other papers focus on direct compression of SNNs. Soures and Kudithipudi propose Deep-LSM, a combination of randomly connected hidden layers and unsupervised winner-take-all layers to capture network dynamics followed by an attention modulated readout layer for classification. The connections between hidden layers and winner-take-all layers are partially trained using STDP. Their SNN model is applied in a first-person video activity recognition task, achieving state-of-the-art performance with >90% memory and operation saving compared to the long-short term memory (LSTM). Based on a single fully-connected layer with the STDP learning rule, Shi et al. propose a soft-pruning method that sets a fraction of the weights to the lower bound during training, effectively achieving >75% pruning. Srinivasan and Roy implement spiking convolutional layers comprising of binary weight kernels which are trained using probabilistic STDP, as well as non-spiking fully-connected layers which are trained using gradient descent. A residual convolutional SNN is proposed, which achieves >20x model compression.

## Programming Tools

Programming Tools have been one of the key components driving development in ANN research, examples of which include Theano (Al-Rfou et al., 2016), TensorFlow (Abadi et al., 2016), Caffe (Jia et al., 2014) and Pytorch (Paszke et al., 2019), MXNet (Chen et al., 2015), Keras (Chollet, 2015). These user-friendly programming tools enable researchers to build and train large-scale ANNs using only basic programming know-how. In comparison, the programming tools for SNNs are rather limited. To the best of our knowledge, only SpiNNaker (Furber et al., 2014), BindsNET (Hazan et al., 2018), and PyNN (Davison et al., 2009) provide a basic programming interface to support simple and small SNN simulations. Generally researchers have to build an SNN from the ground up which can be time-consuming and require significantly more programming know-how. Thus, developing user-friendly programming tools to efficiently deploy a large scale SNN is imperative to the advancement of our field. In this Research Topic, an open-source high-speed SNN simulation framework based on PyTorch has been proposed. SpykeTorch (Mozafari et al.) simulates convolutional SNNs with at most one spike per neuron (rank-order coding scheme), and STDP-based learning rules. Although programming tools for SNNs are still in their infancy, we believe that more research needs to be done so that the training of SNNs may approach the efficiency of training ANNs.

## Neuromorphic Hardwares

Recent advances made in modeling SNNs *in-silico*, as demonstrated by Neurogrid of Stanford University (Benjamin et al., 2014), BrainScales of Heidelberg University (Schemmel et al., 2012), SpiNNaker of University of Manchester, Tianjic of Tsinghua University (Pei et al., 2019), IBM's TrueNorth (Akopyan et al., 2015), and Intel's Loihi (Davies et al., 2018), attest to the great potential of hardware implementation of SNNs. In this Research Topic, Shukla et al. re-model large-scale CNNs so as to mitigate hardware constraints and implement them on the IBM TrueNorth. A CNN used for car detection and counting was demonstrated, with reasonable accuracy compared to a GPU trained CNN but with much lower energy consumption. Bohnstingl et al. implement a learning-to-learn SNN on a neuromorphic chip which accelerates the learning process by extracting abstract knowledge from prior experiences. Other than conventional CMOS circuits, emerging devices such as memristors have also been studied in this Research Topic. Guo et al. propose a STDP-based greedy training algorithm for SNNs to reduce weight levels and enhance robustness toward device non-idealities. They demonstrate online learning on a resistive random access memory (RRAM) system with non-ideal behaviors. Fang et al. propose a generalized swarm intelligence model on SNN: the SI-SNN. SNNs are implemented as agents in swarm intelligence with interactive modulation and synchronization. They implement such neural dynamics on a ferroelectric field-effect transistor (FeFET) based hardware platform to solve optimization problems with high performance and efficiency.

## Conclusions

In conclusion, it is believed that SNNs achieve superior performance in processing complex, sparse, and noisy spatiotemporal information with high power efficiency exploiting neural dynamics in the event-driven regime. Event-driven communication is particularly attractive in enabling energy efficient AI systems with in-memory computing that will play an important role in ubiquitous intelligence. SNN research is ongoing, and much more progress is to be expected in its learning algorithms, benchmarking framework, programming tools, and efficient hardware. Through cross-discipline exchange of ideas and collaborative research, we hope to build a truly energy-efficient and intelligent machine. This Research Topic is but a small step in this direction; we look forward to more disruptive ideas that distinguish SNNs and neuromorphic computing from the mainstream machine learning approaches in the near future.

## Author Contributions

All authors listed have made a substantial, direct and intellectual contribution to the work, and approved it for publication.

### Conflict of Interest

The authors declare that the research was conducted in the absence of any commercial or financial relationships that could be construed as a potential conflict of interest.
